# Selective pattern of motor system damage in gamma-synuclein transgenic mice mirrors the respective pathology in amyotrophic lateral sclerosis

**DOI:** 10.1016/j.nbd.2012.06.016

**Published:** 2012-10

**Authors:** Owen M. Peters, Steven Millership, Tatyana A. Shelkovnikova, Ileana Soto, Lora Keeling, Anthony Hann, Nicholas Marsh-Armstrong, Vladimir L. Buchman, Natalia Ninkina

**Affiliations:** a School of Biosciences, Cardiff University, Museum Avenue, Cardiff, CF10 3AX, UK; b Institute of Physiologically Active Compounds RAS, Russian Academy of Sciences, 1 Severnyj Proezd, Chernogolovka, Russian Federation; c The Solomon H. Snyder Department of Neuroscience, The Johns Hopkins University School of Medicine, Baltimore, MD, USA; d Hugo W. Moser Research Institute at Kennedy Krieger, Baltimore, MD 21205, USA

**Keywords:** ALS, Amyotrophic lateral sclerosis, DRG, Dorsal root ganglion, TDP-43, TAR DNA binding protein-43, FUS/TLS, Fused in sarcoma/translocated in liposarcoma, RGC, Retinal ganglion cell, NF, Neurofilament, pNF, Phosphorylated neurofilament, Neurodegeneration, ALS, Neurofilament, Aggregation, Motor neuron

## Abstract

Amyotrophic lateral sclerosis (ALS) is characterised by substantial loss of both upper and lower motor neuron function, with sensory and cognitive systems less affected. Though heritable forms of the disease have been described, the vast majority of cases are sporadic with poorly defined underlying pathogenic mechanisms. Here we demonstrate that the neurological pathology induced in transgenic mice by overexpression of γ-synuclein, a protein not previously associated with ALS, recapitulates key features of the disease, namely selective damage and loss of discrete populations of upper and lower motor neurons and their axons, contrasted by limited effects upon the sensory system.

## Highlights

► Selective loss of brainstem motor neurons and atrophy of cortical motor regions. ► Selective loss of the largest caliber motor axons in peripheral nervous system. ► Preservation of sensory neurons and sensory function in γ-synuclein transgenic mice.

## Introduction

Amyotrophic lateral sclerosis (ALS) is characterised by the severe deficiency of both upper and lower motor neuron function, with sensory and cognitive systems less affected. Though ALS-associated genetic alterations have been identified in several loci (DeJesus-Hernandez et al., 2011; Deng et al., 2011; Kwiatkowski et al., 2009; Maruyama et al., 2010; Renton et al., 2011; Rosen et al., 1993; Sreedharan et al., 2008; Vance et al., 2009) the vast majority of clinical cases are sporadic, with scant evidence of common risk factors (Chio et al., 2009; van Es et al., 2009; Wain et al., 2009). In order to fully understand and ultimately treat ALS it is essential we broaden our spectrum of knowledge beyond the disease's genetic background, and consider novel factors that might contribute to its underlying pathology. γ-Synuclein, a cytosolic protein abundant in the perikarya, presynaptic terminals and particularly within the axons of select populations of CNS and PNS neurons (Buchman et al., 1998), is a close relative of α-synuclein — a protein robustly linked to the idiopathic (Edwards et al., 2010; Pankratz et al., 2009; Satake et al., 2009; Simon-Sanchez et al., 2009) and familial (Chartier-Harlin et al., 2004; Ibanez et al., 2004; Kruger et al., 1998; Polymeropoulos et al., 1997; Singleton et al., 2004; Zarranz et al., 2004) forms of neurodegenerative diseases known as synucleinopathies. Intracellular accumulation, oligomerisation and aggregation of α-synuclein are arguably causes of pathology, with the presence of inclusions formed from fibrillated α-synuclein being a hallmark of these diseases. γ-Synuclein shares this ability to aggregate (Park and Lansbury, 2003) and, though not identified as a constituent of any common lesions such as Lewy bodies, has been described as a component of atypical inclusion bodies in human neurodegenerative conditions (Galvin et al., 1999, 2000; Surgucheva et al., 2002) and rodent axonopathy models (Nguyen et al., 2011; Wang et al., 2004). Changes in γ-synuclein expression and its pathological accumulation in specific regions of the optic nerve (Buckingham et al., 2008; Nguyen et al., 2011; Soto et al., 2008; Surgucheva et al., 2002) are emerging as possible contributing factors in glaucoma. Polymorphisms in the γ-synuclein locus have recently been associated with human diffuse Lewy body disease (Nishioka et al., 2010). Taken together with high abundance of γ-synuclein in motor neurons and particularly their axons (Buchman et al., 1998; Ninkina et al., 2003, 2009), these observations raised a possibility that dysfunction of this protein might be directly involved in the pathogenesis of some forms of motor neuron diseases. This prompted us to investigate if certain characteristic features of ALS pathology were recapitulated in the mouse model of γ-synucleinopathy.

## Materials and methods

### Experimental animals

The generation of Thy1mγSN mice on a pure C57Bl6J genetic background has been described previously (Ninkina et al., 2009). All animal works were carried out in accordance with the United Kingdom Animals (Scientific Procedures) Act (1986).

### Inverted grid test

To assess limb muscle strength a mouse was placed onto a 30 cm by 30 cm square mesh consisting of 5 mm squares of 0.5 mm diameter wire. The grid was slowly rotated to inverted position and held 30 cm above the surface of a thick layer of bedding material. If a mouse fell from the inverted grid earlier than the maximum test time of 1 min the latency to fall was noted and after an appropriate rest in the home cage the test was repeated. The best time from three attempts was included in the statistics. At minimum 9 mice were tested in each group.

### Analysis of hind paw withdrawal threshold following tactile stimulation

Mice were placed in a plastic cage with a mesh base composed of 5 mm squares of 0.5 mm diameter wire and allowed 30 minutes to habituate. Tests were only carried out when the animal was still, with all four paws on the cage surface. Von Frey hairs (SOMEDIC, Sweden) of varying force (0.12 g–6.20 g) were applied from beneath the animal to the plantar surface, taking care to avoid the footpads, for approximately 2 seconds. Each hair was applied ten times. Increasing force of von Frey hair was used until either the highest force hair or the 100% withdrawal threshold was met. Responses considered valid were the lifting of paws from surface of the grid or the spanning of digits upon stimulation. The latter response was frequently observed when the lowest force hairs were used. A minimum of 7 animals were tested in each group.

### Sequential protein fractionation

Protein from cytosolic fractions of motor and sensory cortices of 12-month old mice were fractionated as described previously (Ninkina et al., 2009).

### Quantitative Western blotting

Previously described protocols were used for Western blotting (Buchman et al., 2002). Protein levels were quantified using Cy3- or Cy5-conjugated secondary antibodies (Invitrogen, CA) and FluorChem Q MultiImage III system (Cell Biosciences, CA).

### Histological techniques

For Nissl staining sections were incubated in 0.5% cresyl fast violet solution (Sigma-Aldrich, MO) and differentiated in acidified ethanol. Nissl stained cranial motor neuron cell bodies and DRG sensory cell bodies were identified by their large cell body densely stained with Nissl granules, clear nuclear envelope and intensely stained nucleolus. Every 10th section of the abducens, facial and trigeminal motor nucleus, and 6th section of L4/5 DRG was sampled. The total number of cells for each population was estimated using the cell fractionator technique. Immunostaining was carried out as described previously (Ninkina et al., 2003, 2009) using Elite plus kits (Vector Laboratories, CA) and 3,30-diaminobenzidine as a substrate (Sigma-Aldrich, MO) or Alexa Fluor-conjugated secondary antibodies (Invitrogen, CA). Expression of RNA and proteins in the mouse retina whole mounts was carried out as described previously (Nguyen et al., 2011; Surgucheva et al., 2002).

### Primary antibodies

Antibodies against γ-synuclein (rabbit polyclonal SK23, affinity purified), NF-L (mouse monoclonal, clone DA2, Cell Signaling, MA), NF-M (mouse monoclonal, clone NN18, Sigma, MO), NF-H (rabbit polyclonal, Sigma, MO), α-tubulin (mouse monoclonal, clone DM 1A, Sigma, MO), peripherin (rabbit polyclonal, Chemicon, MA), myelin basic protein (mouse monoclonal, clone MAB387, Chemicon, MA), GFAP (rabbit polyclonal, Sigma-Aldrich, MO) and NeuN (mouse monoclonal, Chemicon, MA) were used as described previously (Ninkina et al., 2009). Biotinylated lectin ricin communis agglutinin 1 was used for detection of microglia as suggested by the manufacturer (Vector). For retina studies, antibodies against γ-synuclein (rabbit polyclonal to C-terminal 16 amino acids, affinity purified) and pNF (SMI31, Covance, NJ) were used as described previously (Nguyen et al., 2011). In situ hybridisation to γ-synuclein mRNA was carried as described previously (Soto et al., 2008).

### Transmission electron microscopy

Nerves were submerged in cold 2% paraformaldehyde, 2% gluteraldehyde in 0.1 M Sorenson's PB, post-fixation in 1% osmium tetraoxide, block stained in 0.5% uranyl acetate and embedded in araldite (Agar Scientific, UK). Semi-thin section (0.6 μm) were collected and stained with 1% toluidine blue, 1% borax in dH_2_O. Ultra-thin sections (0.095 μm) were mounted on a 0.8% pioloform film coated copper slot grids (Agar Scientific, UK) and counterstained with 2% uranyl acetate and Reynold's lead citrate. Axon area measurements were made using ImageJ analysis software (NIH, MD).

### Morphometric analysis of sciatic nerve and spinal nerve roots

Myelinated A-fibres and unmyelinated C-fibre were counted within a randomly selected field at 2000 × magnification. All A-fibres were counted recording their morphological condition, with fibres classed as either normal or damaged. Healthy fibres were defined as those with both axons and myelin sheath intact. Unhealthy fibres had dense, vacuous or absent axons, substantial abnormalities in the myelin sheath, or a combination of both. Damaged axons fully enveloped by phagocytes were not counted as the digestive process of these cells made accurate quantification impossible. All counts were normalised to a 100 μm^2^. Axon area measurements were made using ImageJ analysis software (NIH, MD).

## Results

### Motor neuron populations are selectively vulnerable to the overexpression of γ-synuclein

**Fig. 1:**
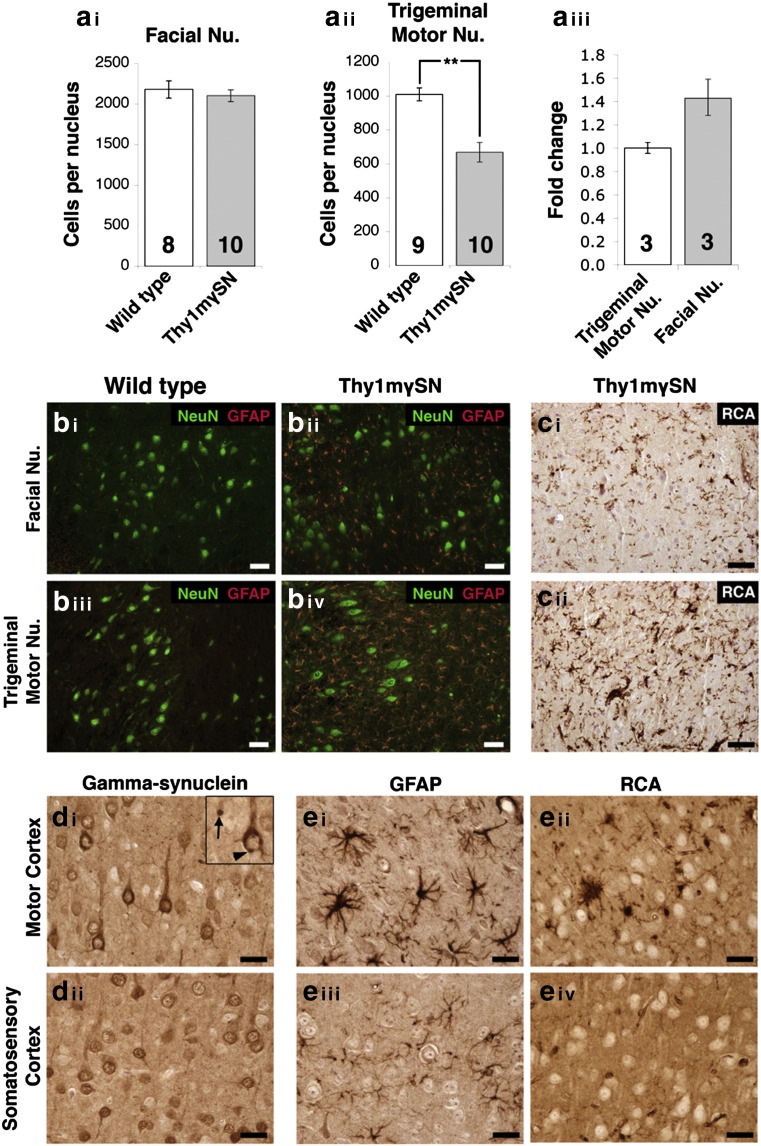
Cranial motor regions of γ-synuclein transgenic mice show a selective pattern of damage. Quantification of motor neurons (mean ± SEM, **p < 0.005) in facial (a_i_) and trigeminal motor (a_ii_) nuclei of 12-month old wild type and Thy1mγSN mice. The number of nuclei assessed is shown. Levels of transgene expression in the motor trigeminal and facial nuclei of presymptomatic 4-month old Thy1mγSN mice measured by quantitative real-time RT-PCR, using RNA extracted from fresh tissue microdissected from 1 mm brain slices of individual animals (a_iii_). The number of animals from which cranial nuclei were dissected bilaterally is shown. Immunofluorescent staining of the wild type (b_i_, b_iii_) and Thy1mγSN (b_ii_, b_iv_) mouse brain sections with antibodies against NeuN (green) and GFAP (red) shows substantially higher level of astrogliosis in the trigeminal motor nucleus (b_iv_) than in facial nucleus (b_ii_) of transgenic mice. Ricin communis agglutinin 1 (RCA) lectin labeling (c) revealed a similar trend for microgliosis, with a considerably higher level in the trigeminal motor nucleus (c_ii_) than facial nucleus (c_i_). Various abnormal γ-synuclein-positive structures (d_i_ insert, arrow = axonal spheroid, arrowhead = perikaryal cytoplasmic inclusion) were distinctly more abundant in the Thy1mγSN motor cortex (d_i_) than the somatosensory cortex (d_ii_), as were activated astrocytes (e_i_, e_iii_) and microglial cells (e_ii_, e_iv_). Scale bars: b, c = 50 μm, d, e = 25 μm.

We have previously demonstrated that mice with Thy1 promoter driven-overexpression of murine γ-synuclein in many central and peripheral neuron populations (herein Thy1mγSN mice) develop a progressive, age-dependent motor dysfunction, correlating with the formation of γ-synuclein-containing inclusion bodies, which are negative for TDP-43, FUS, α-synuclein, tau and only occasionally ubiquitin-positive, and substantial depletion of motor neuron pools at all levels of the spinal cord (Ninkina et al., 2009). In addition to severe loss of spinal motor neurons, a typical feature of ALS is the loss of lower motor neuron in selective brainstem populations (Wijesekera and Leigh, 2009). Therefore we stereologically counted neurons in cranial motor nuclei of severely affected 12-month old Thy1mγSN and age-matched healthy wild type mice. Significant neuron loss, comparable with that seen in the spinal cord was observed in the trigeminal motor nucleus (33.9% loss, *p* = 0.0012, Mann–Whitney *U*-test), whilst a normal complement of motor neurons was found in the facial and abducens nuclei of transgenic mice (Fig. 1a, A.1a–g and Table A.1). Consistently, astrogliosis and microgliosis, which are commonly associated with neurodegeneration, were most prominent in the affected nucleus (Fig. 1b, c). Notably, the selective nature of γ-synuclein induced pathology did not coincide with substantial differences in transgene expression between nuclei, with Thy1mγSN transgene expression found to be slightly higher in the resistant facial nucleus than in the sensitive motor trigeminal nucleus (Fig. 1a_iii_, A.1h–k). Evidence of selective neuronal dystrophy was also observed in the cortex, with perikaryal and axonal accumulation of γ-synuclein (Figs. 1d, A. 2a), accumulation of detergent-insoluble species (Fig. A. 2b), and substantial gliosis observed within motor regions (Figs. 1e, A. 2c, d), however all to a lesser degree in the somatosensory regions. These results suggest that γ-synuclein overexpression is damaging to select lower and upper motor neuron populations, a hallmark feature of human ALS (Wijesekera and Leigh, 2009) also detected in some rodent models of ALS (Ferrucci et al., 2010).

### Severe damage and loss of myelinated axons in peripheral nerves of Thy1mγSN mice correlate with the development of motor deficits

**Fig. 2:**
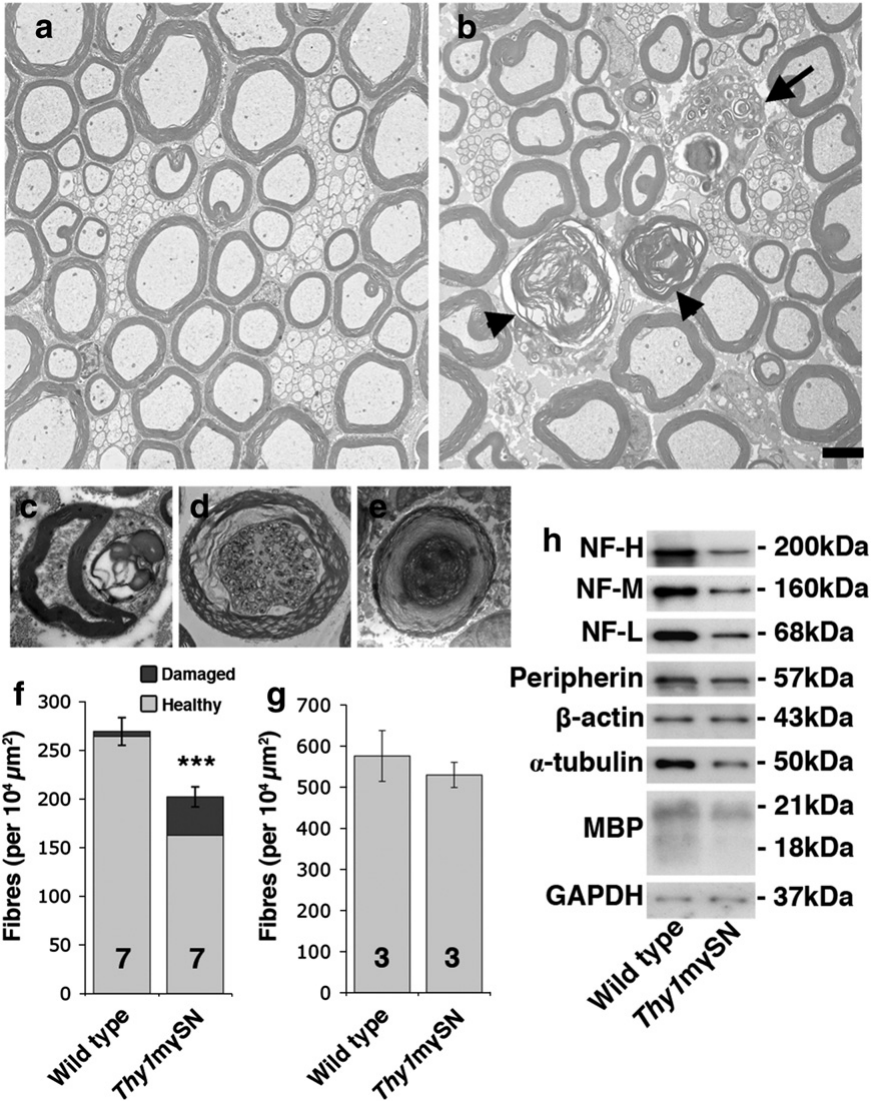
Severe damage of fibres in the sciatic nerves of mice overexpressing γ-synuclein. Electron micrographs of transverse sections of the sciatic nerve of 12-month old mice (a–e). Abnormal myelinated fibres are abundant in Thy1mγSN (b) but not wild type mice (a). Representative images of fibres with damage to their myelin sheath (c), damaged axon (d) or a highly degraded multilamellar structure (e) are shown. Quantification of total, healthy and damaged myelinated fibres (f) and total C-fibres (g) in the sciatic nerve of wild type and Thy1mγSN mice (mean ± SEM, ***p < 0.001, Mann–Whitney *U*-test). Representative Western blot (h) shows that levels of several structural proteins are decreased in the sciatic nerves of 12-month old Thy1mγSN mice. For full quantification see Table A. 2. Scale bar: a, b = 3 μm.

ALS-associated motor neuron cell body loss is generally accompanied by the degeneration of their processes. Quantitative Western blotting revealed changes in the levels of several major axonal cytoskeletal proteins in the sciatic nerve of Thy1mγSN mice with severe motor phenotype. Levels of all three neurofilament proteins and α-tubulin were substantially reduced compared to the nerves of age-matched wild type mice, whereas peripherin and actin levels remained unchanged (Fig. 2h and Table A. 2). Levels of the 18 kDa and 21 kDa isoforms of myelin basic protein, principal components of the axonal myelin sheet, were also reduced.

Substantial reductions in the levels of cytoskeletal and myelin markers suggested a gross change in sciatic nerve morphology. Indeed, a sparser distribution and deterioration of myelinated axons was clearly evident in severely affected Thy1mγSN mice (Fig. 2b). A substantial number of the myelinated fibres were noted to have a highly abnormal morphology, including damage to their myelin sheath (Fig. 2c–e). Widespread invasion by phagocytes containing whirls of digested myelin (Fig. 2b) indicated active Wallerian-like degeneration of the nerve. Quantitative analysis revealed a significant reduction in the total number of the myelinated fibres in the sciatic nerve of Thy1mγSN mice (24.9% less fibres than in wild type mice, *p* = 0.0005, Mann–Whitney *U*-test) and, when classified by morphological condition, a prominent loss of healthy fibres (38.6%, *p* < 0.0001, Mann–Whitney *U*-test) was accompanied by a dramatic ~ 8 times increase in damaged fibres (Fig. 2f). No loss of unmyelinated sensory C-fibres was observed in the sciatic nerve of symptomatic Thy1mγSN mice (Fig. 2g). The presence of structural changes in the sciatic nerve of Thy1mγSN mice correlated with the severity of motor phenotype. Only a minor reduction in the levels of structural proteins, slight alteration of the morphology and no loss of fibres were observed in transgenic mice with a mild deficit of motor function (Tables A. 2 and A. 3).

### No progressive loss of tactile sensory perception in Thy1mγSN mice

**Fig. 3:**
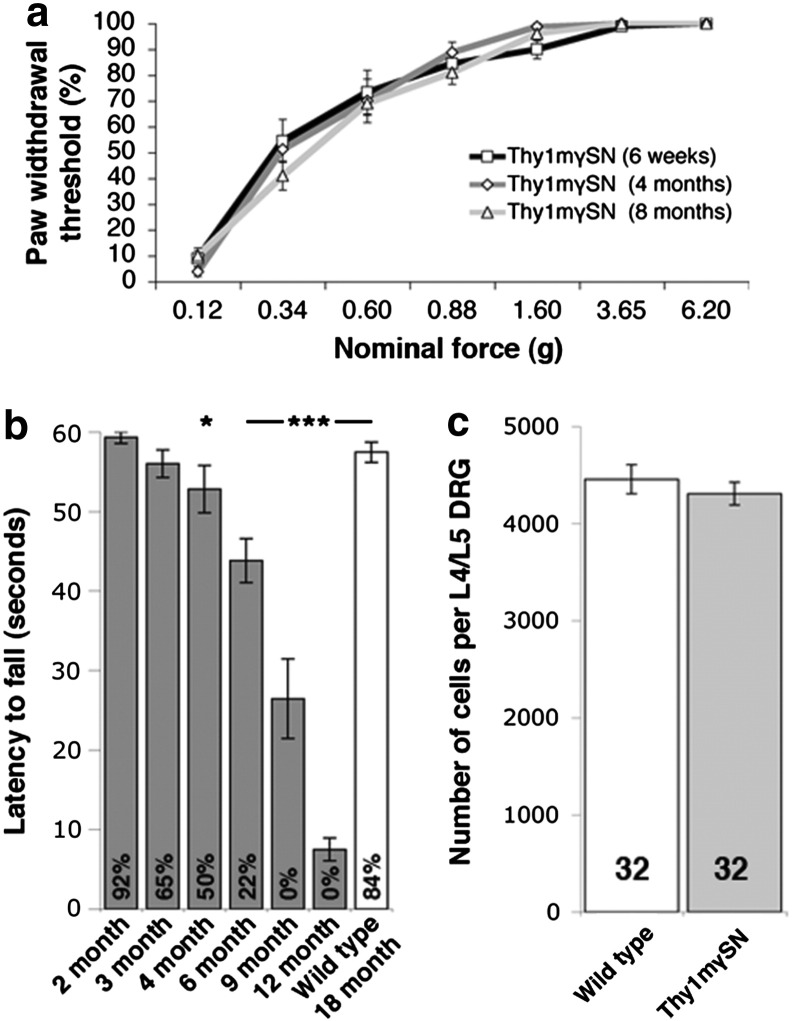
The progressive motor deficit seen in Thy1mγSN mice is not accompanied by changes in tactile sensitivity. Inverted grid test and response to tactile stimulation of the hind plantar surface (mean ± SEM, *p < 0.05, ***p < 0.001 Mann–Whitney *U*-test). Thy1mγSN mice develop no impairment in response to tactile stimuli between 6 weeks, 4 months and 8 months (a). In contrast the ability of Thy1mγSN mice to hang from an inverted grid substantially diminished with age (b). Inset number denotes percentage of animals able to successfully complete the 60-second test. The number of neurons in L4/5 dorsal root ganglia (mean ± SEM) of severely affected 12-month old Thy1mγSN mice and healthy age-matched wild type controls (c).

Motor dysfunction is widely accepted as the main clinical feature of ALS, while functional and morphological changes in the sensory systems of patients and rodent models of the disease are less common and typically modest (Guo et al., 2009; Hammad et al., 2007; Isaacs et al., 2007; Kawamura et al., 1981; Pugdahl et al., 2007). Myelinated fibres in the sciatic nerve include both afferent and efferent neuron projections, and therefore it was important to investigate if the damage observed in Thy1mγSN mice had a debilitating effect on both the motor and sensory system. In contrast to the age-correlated decline of motor function in Thy1mγSN mice, previously revealed in the rotarod tests (Ninkina et al., 2009) and here illustrated by results of the inverted grid test (Fig. 3b), assessment of hind-paw withdrawal threshold following tactile stimulation of the plantar surface revealed no progressive deficit between the evoked response of 6-week, 4-month and 8-month old animals (Fig. 3a). Histological assessments of ganglia also demonstrated sensory system preservation—neither γ-synuclein inclusions nor gliosis (Fig. A. 3) was observed and stereological counting revealed no loss of sensory neurons in the L4/5 dorsal root ganglia in 12-month old Thy1mγSN mice (Fig. 3c), despite the high level of transgene expression in these ganglia (Ninkina et al., 2009).

### Myelinated fibres are more affected in ventral than dorsal roots of the spinal nerves of 12-month old Thy1mγSN mice

**Fig. 4:**
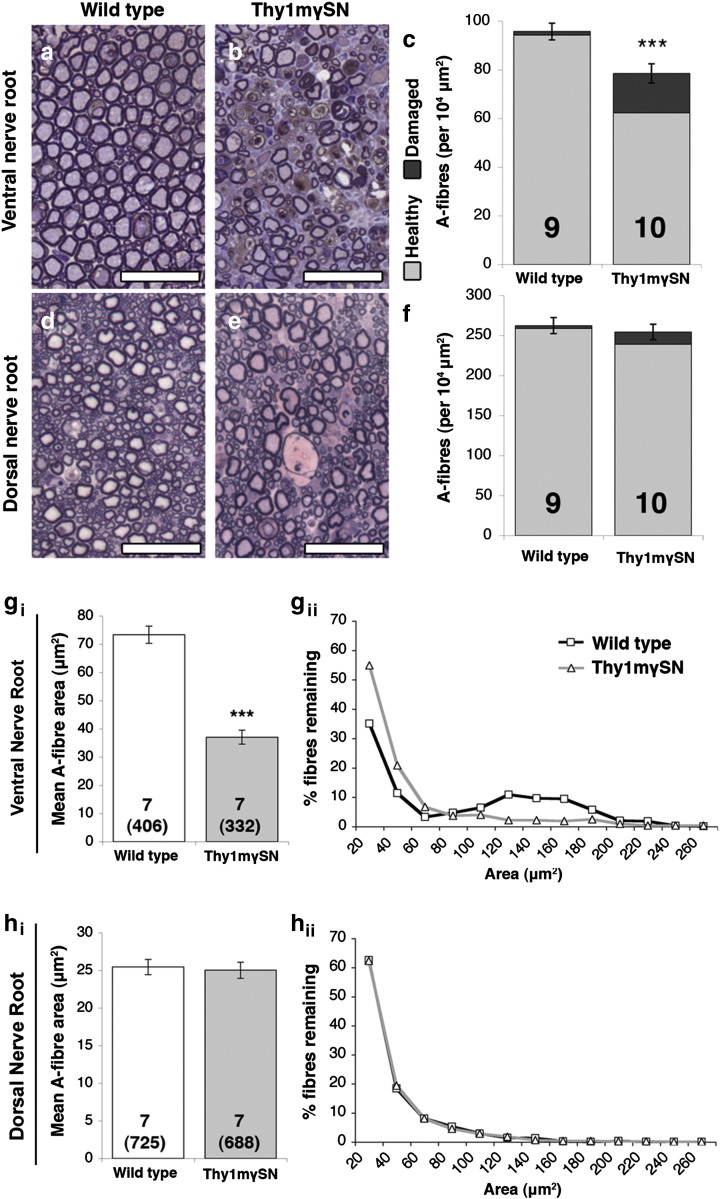
The population of motor fibres entering the sciatic nerve are both damaged and diminished. Representative toluidine blue stained transverse semi-thin sections through ventral (a, b) and dorsal (d, e) nerve roots of 12-month old wild type (a, d) and Thy1mγSN (b, e) mice. Quantification of total, healthy and damaged myelinated fibres in L4–L5 ventral (c) and dorsal (f) nerve roots of 12-month old mice (mean ± SEM, ***p < 0.001 Mann–Whitney *U*-test). The size (mean area ± SEM, ***p < 0.001, Mann–Whitney *U*-test) and size distribution of motor nerve fibres is significantly smaller in the ventral nerve root of 12-month old severely affected Thy1mγSN than age-matched wild type mice (g_i_), due to a distinct shift towards smaller classes of fibre surviving (g_ii_). The mean area and size distribution of sensory fibres do not differ between genotypes (h_i_, h_ii_). The number of animals analysed and total number of fibres measured (in brackets) are shown. Scale bars: a–d = 50 μm.

To further investigate the pattern of myelinated fibre degeneration induced by γ-synuclein overexpression, we assessed the morphology of the discrete pools of sensory and motor fibres located in lumbar dorsal and ventral nerve roots respectively. The orderly distribution of myelinated motor fibres seen in ventral roots of wild type mice (Fig. 4a) was substantially disrupted in Thy1mγSN mice (Fig. 4b). Quantification revealed a significant loss of myelinated motor fibres and high proportion of severely damaged fibres (Fig. 4c, Table A. 4). Similar to changes found in the sciatic nerve, we observed an 18% decrease in numbers of total fibres, 33.9% decrease in healthy fibres and 10.8 times increase in numbers of damaged fibres contained in the ventral roots of Thy1mγSN mice compared to the corresponding values of wild type mice (*p* < 0.001 for all, Mann–Whitney *U*-test). These pathological changes were accompanied by extensive phagocyte infiltration (Fig. A. 4). It has been demonstrated that in the peripheral nerves of ALS patients (Kawamura et al., 1981) and rodent models of the disease (Bruijn et al., 1998; Kong and Xu, 1998; Shan et al., 2010) the largest caliber myelinated motor fibres degenerate more readily than the smaller class of slow-firing fibres. Analysis of the size and size distribution of remaining myelinated motor fibres in the ventral roots of Thy1mγSN mice showed a substantial 49.5% decrease (*p* < 0.001, Mann–Whitney *U*-test) in the mean myelinated fibre size compared to wild type mice, caused by a disproportionate loss of the largest caliber fibres (Fig. 4g). In contrast, the vast majority of fibres in the dorsal roots of Thy1mγSN mice were healthy and equal in number (Fig. 4f and Table A. 4), size and size distribution (Fig. 4h) to those of wild type animals, though some abnormal myelinated fibres (Fig. 4f) and phagocytes (Fig. A. 4b) were observed.

**Fig. 5:**
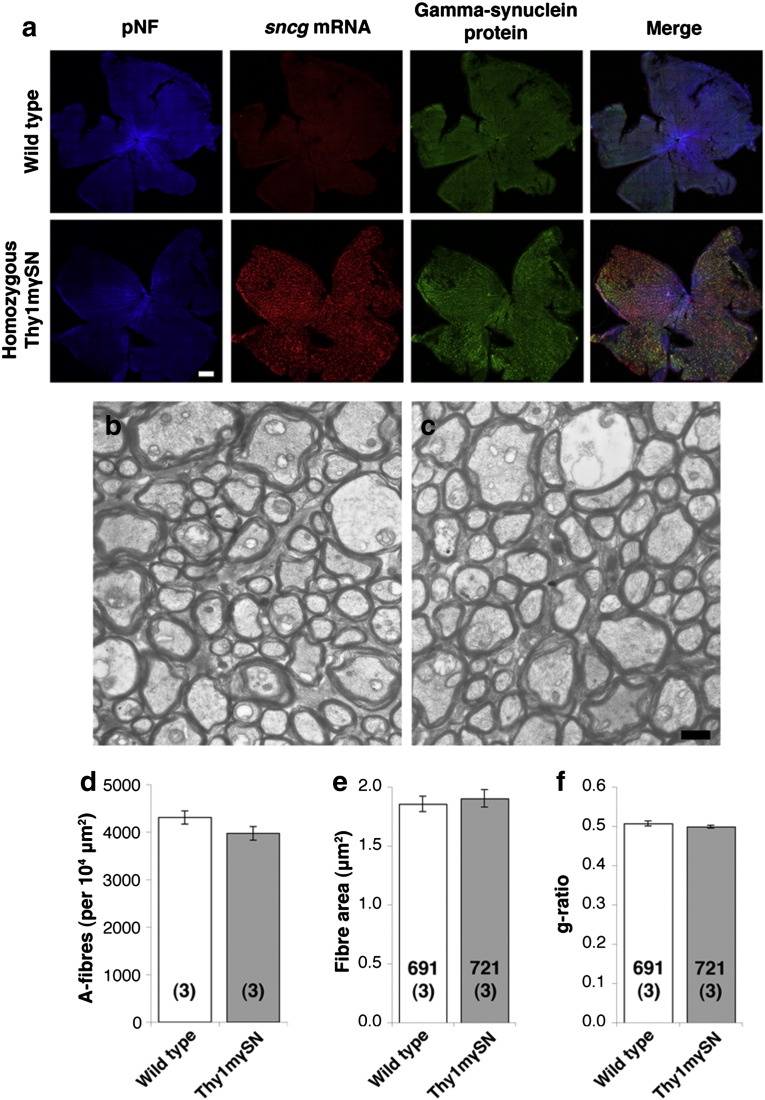
Retinal ganglion cells and their axons in the optic nerves do not degenerate after overexpression of γ-synuclein in Thy1mγSN mice. Detection of γ-synuclein mRNA expression by *in situ* hybridisation (red), protein expression by immunostaining with antibodies against γ-synuclein (green) and damaged RGCs by immunostaining with antibody against phosphorylated neurofilaments (blue) in whole mounted retina of 12-month-old Thy1mγSN and wild type mice (a). Representative electron micrographs of transverse sections of the optic nerve of 12-month old wild type (b) and *Thy1*mγSN mice (c), and quantification of total number of myelinated fibres (d), mean fibres area (e) or g-ratio (f) of fibres (mean ± SEM, Mann–Whitney *U*-test). The total number of fibres measured and animals analysed (in brackets) are shown. Scale bars: a = 500 μm, b, c = 1 μm.

Further evidence of selective sensitivity of various neuronal populations to γ-synuclein overexpression came from studies of the retina of 12-month old Thy1mγSN mice. Despite high levels of transgenic RNA and protein expression in the retinal ganglion cells, RGCs (Fig. 5a), no evidence of cell bodies or axon degeneration was detected. There was no obvious increase in the number of RGCs with somatic accumulation of phosphorylated neurofilament (Fig. 5a), a marker of damaged RGCs (Buckingham et al., 2008; Nguyen et al., 2011). Quantification of sensory axons in ultra-thin sections of optic nerve revealed no loss (Fig. 5d, Table A. 5), and no changes in axon size (Fig. 5e) or myelin sheath thickness (denoted by g-ratio) were detected (Fig. 5f). Taken together these results suggest preservation of this sensory system in Thy1mγSN mice.

## Discussion

The pattern of neurodegeneration developed in the nervous system of Thy1mγSN mice recapitulates several notable pathological features common to sporadic and familial forms of ALS. These include a progressive decline in motor function caused by severe damage to motor fibres, in particular those of largest caliber, in peripheral nerves and substantial loss of selective motor neuron populations; preservation of sensory neurons morphology and function, including those of the visual system; prominent neuroinflammation restricted to areas where significant damage of motor neurons is evident. The absence of TDP-43 or FUS pathology in Thy1mγSN mice indicates that the development of γ-synuclein pathology is most likely a downstream event in pathogenesis of diseases triggered by dysfunction of the former proteins. Although γ-synuclein is not currently recognised as a major contributing factor in human neurodegenerative diseases, there is a growing body of evidence that under certain conditions this protein plays a role in axonal dysfunction in both humans (Galvin et al., 1999, 2000), and mice (Nguyen et al., 2011; Ninkina et al., 2009; Soto et al., 2008; Wang et al., 2004). Our data now suggests that disruption of normal γ-synuclein metabolism, followed by its aggregation, might be a contributing factor in the pathogenesis of ALS, a hypothesis supported by our recent finding (unpublished observations, manuscript submitted) that revealed accumulation and aggregation of γ-synuclein in a subset of ALS cases. The Thy1mγSN mouse line may thus provide a useful tool in dissecting the molecular involvement of γ-synuclein in certain types of neurodegenerative diseases.
