# Supplementary material for: Selective pattern of motor system damage in gamma-synuclein transgenic mice mirrors the respective pathology in amyotrophic lateral sclerosis
Source: Neurobiol Dis. 2012 Oct;48(1):124–31. doi: 10.1016/j.nbd.2012.06.016 (PMC3457776; doi:10.1016/j.nbd.2012.06.016)
Supplement: Supplementary file 1 — Supplementary materials. [file mmc1.doc]

**APPENDIX**

**Figures**

**
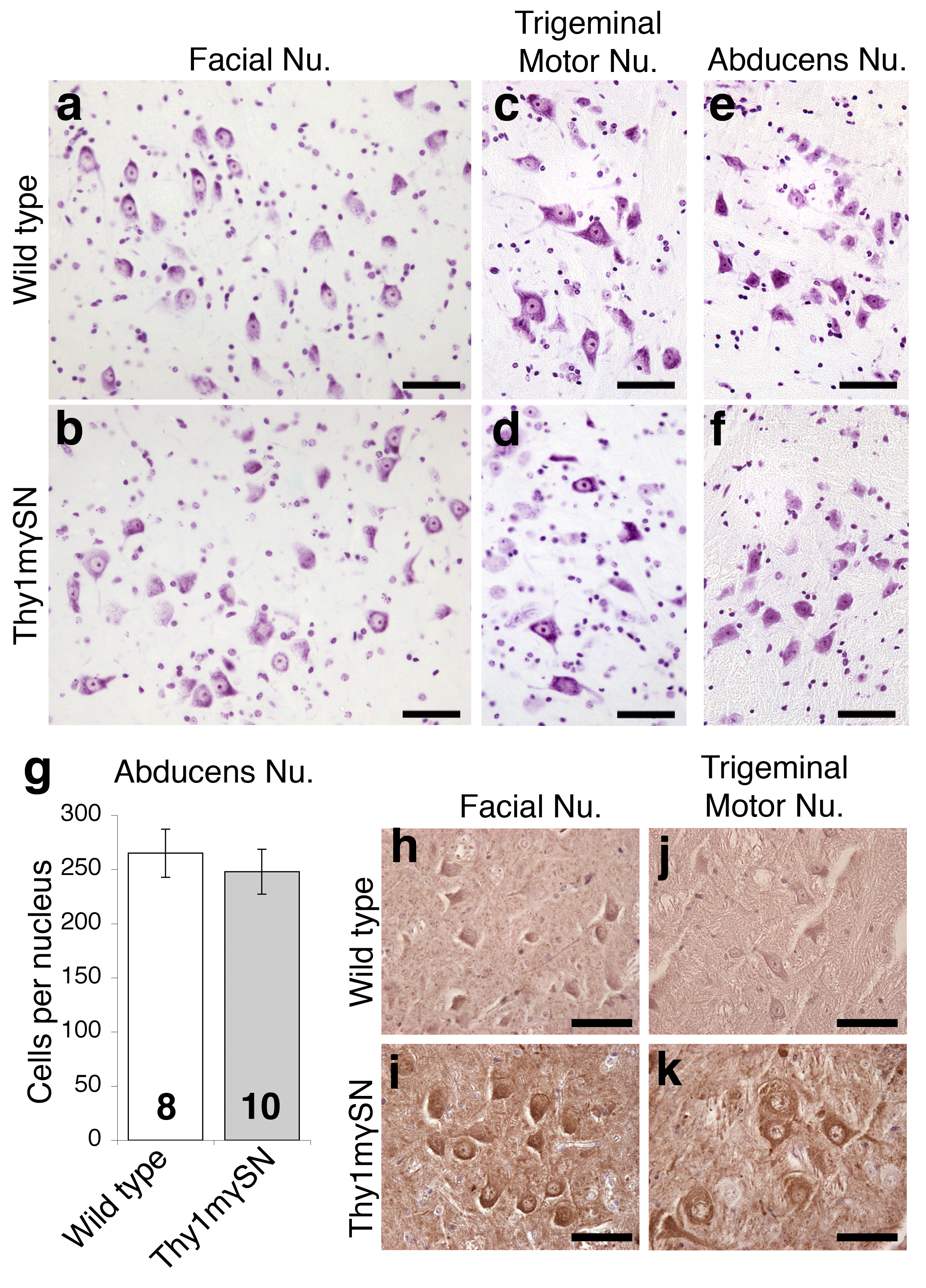
**

**Figure A. 1: Differential sensitivity of cranial motor neuron populations to** γ**-synuclein induced toxicity.** Representative images of Nissl stained brain sections from twelve-month old Thy1mγSN mice illustrate no loss of motor neurons in the facial (b) or abducens (f) nuclei when compared to those of age-matched wild type mice (a and e, respectively). However, significantly fewer motor neurons were observed in the trigeminal motor nucleus of Thy1mγSN (d) than wild type (b) mice. Bar chart (g) shows the number of motor neurons (mean±SEM) in the abducens nucleus of 12-month old wild type and Thy1mγSN mice. The number of nuclei assessed is shown. For quantification of neurons in the facial and trigeminal motor nucleus see main Fig. 1. This selective degeneration is not due to variations in transgene expression. γ-synuclein immunostaining of the facial (i) and motor trigeminal nuclei (k) of 12-month old Thy1mγSN mice showed comparable levels of neuron cell body staining between the nuclei, and in both cases levels were substantially higher than that seen in wild type tissue (h and j respectively). All scale bars = 50μm.

**
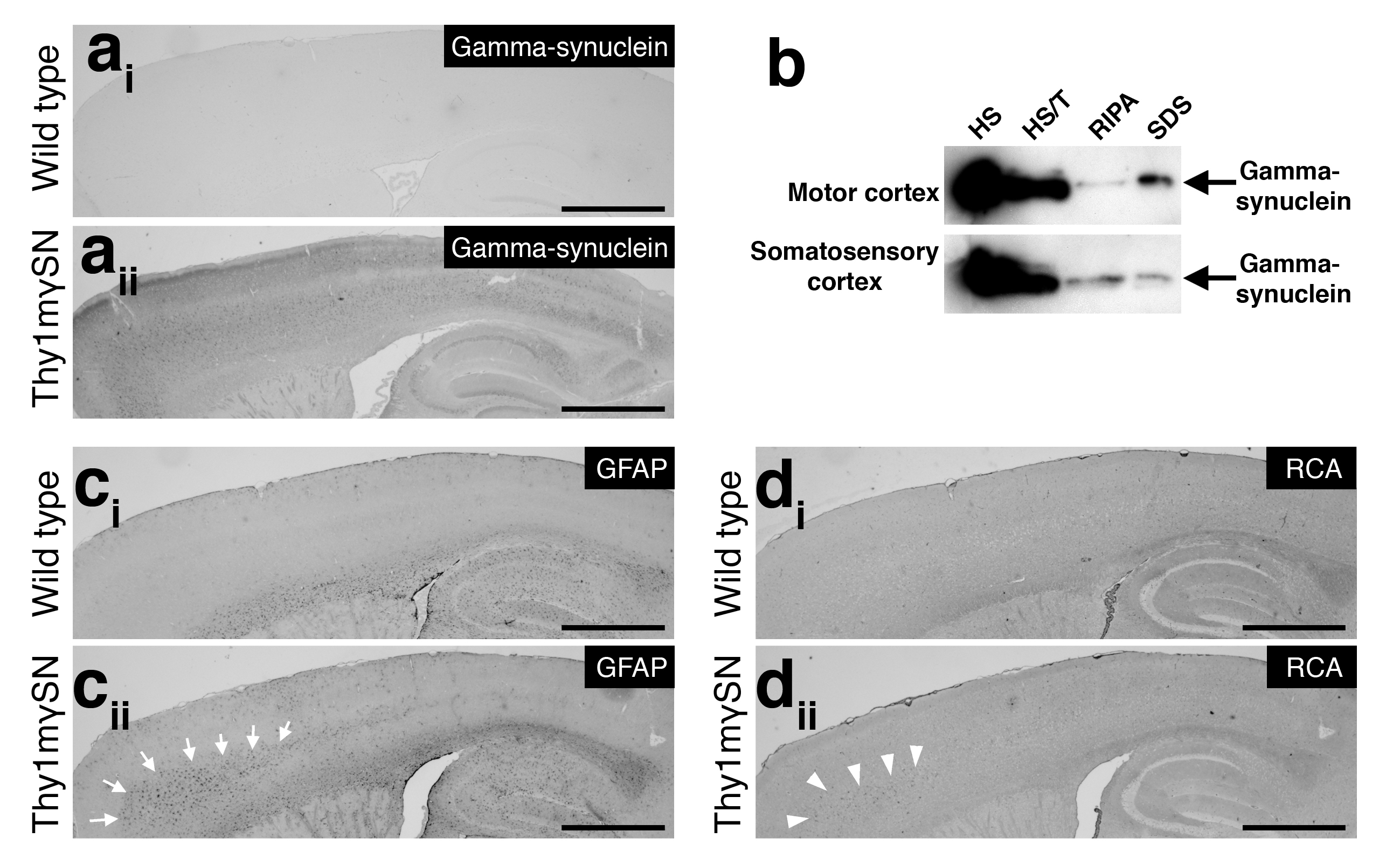
Figure A. 2: Differential cortical pathology in response to γ-synuclein induced toxicity.** Immunostained sagittal brain sections from 12-month old wild type (a_i_, c_i_, d_i_) and homozygous Thy1mγSN mice (a_ii_, c_ii_, d_ii_). Western blot analysis of γ-synuclein in high salt (HS), HS/Triton X-100 (HS/T), RIPA- and SDS-soluble fractions of motor and somatosensory cortices (b). Negligible levels of γ-synuclein (a_i_), astro- (c_i_) and microgliosis (d_i_), were detected in the cortices of wild type mice. Perikaryal and axonal γ-synuclein are found throughout internal and external pyramidal cell layers of age-matched Thy1mγSN mice (a_ii_). Abnormal inclusions (See Fig 1d_v_) and insoluble forms of γ-synuclein (b) however were most frequent in the motor region. Accordingly substantial astrogliosis (c_ii_) and microgliosis (d_ii_) were seen in the motor cortex but not in the somatosensory region. Arrows = activated astrocytes, arrowheads = activated microglia. Scale bars a, c, d = 1mm.

**
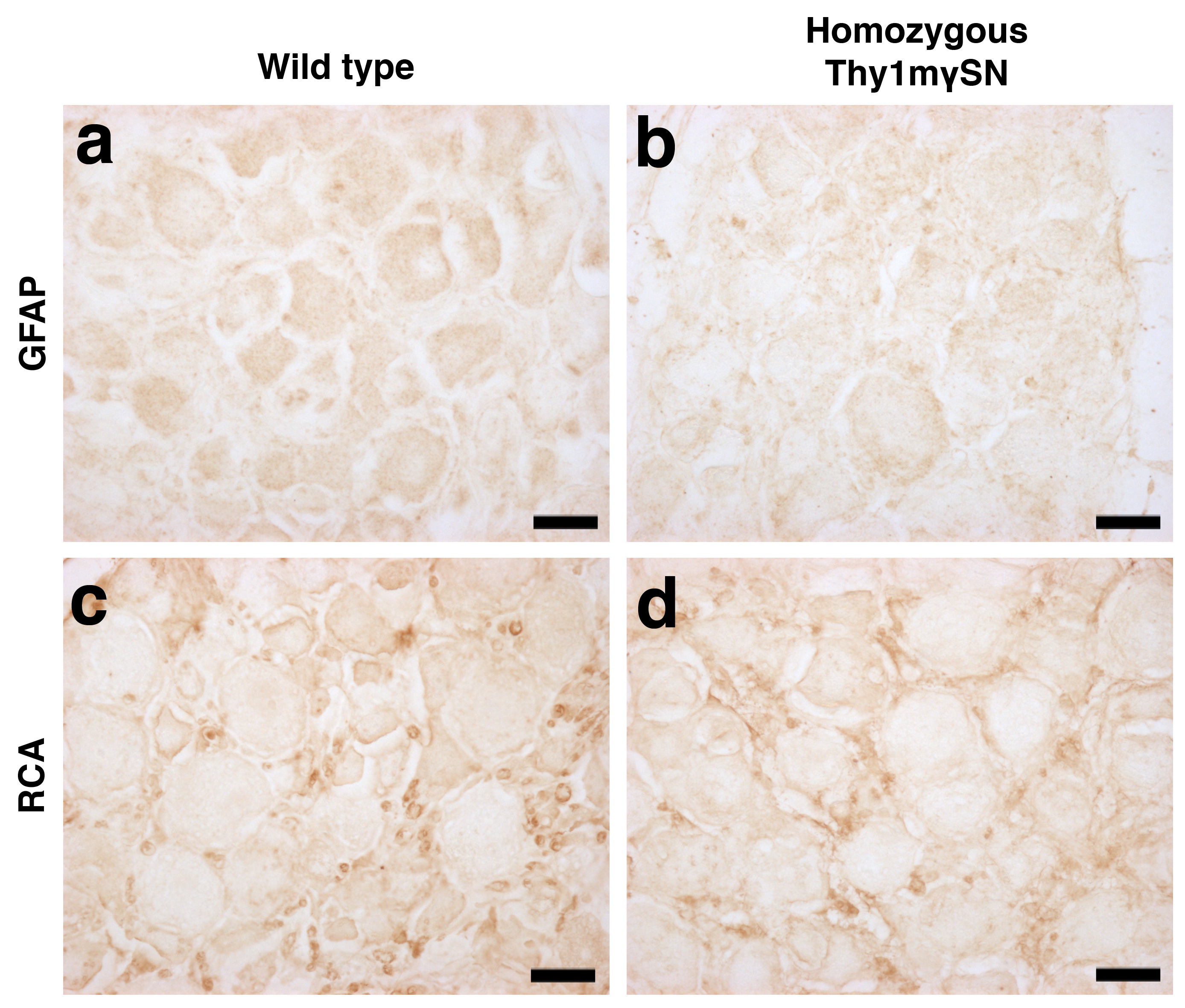
**

**Figure A. 3: Overexpression of γ-synuclein in the dorsal root ganglia results in neither inclusion body formation nor gliosis.** Representative micrographs of lumbar dorsal root ganglia of twelve-month old wild type (a, c) and Thy1mγSN mice (b, d) immunostained with antibodies against GFAP (a, b) and RCA lectin labeling (c, d). Scale bars = 25μm.

**
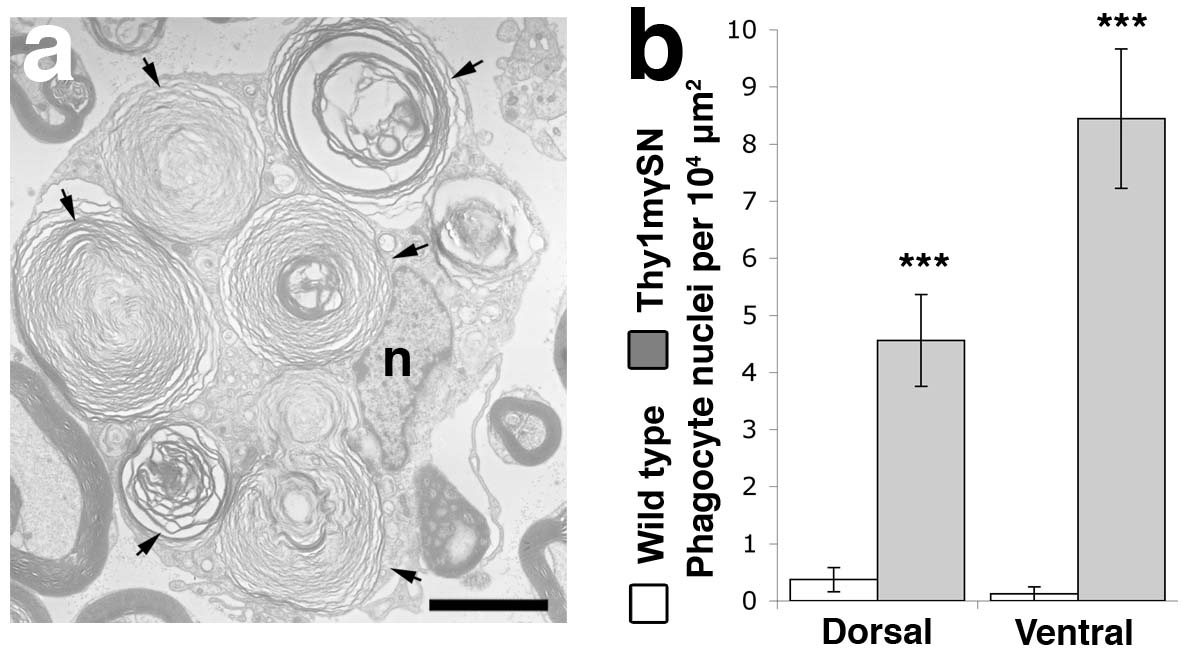
**

**Figure A. 4: Extensive phagocytosis in the spinal nerve roots of severely symptomatic Thy1mγSN mice.** Electron-micrograph of phagocyte containing whirls of myelin, found in lumbar ventral nerve root (a). Arrows indicate phagocytosed myelin, ‘n’ indicates the nucleus. The number of phagocytic cells (mean±SEM, *** p<0.001, Mann-Whitney U-test) found within the sensory and motor nerve roots of Thy1mγSN mice (b). Scale bars a-d = 50μm.

Tables

| **Abducens nucleus** | **n** | **Total cell bodies per nucleus** |
| --- | --- | --- |
| Wild type | 8 | 265.0 ± 22.3 |
| Thy1mγSN | 10 | 248.0 ± 20.7 |
|  |  | p=0.9645 |
|  |  |  |
| **Facial nucleus** | **n** | **Total cell bodies per nucleus** |
| Wild type | 8 | 2180.0 ± 105.9 |
| Thy1mγSN | 10 | 2102.0 ± 71.7 |
|  |  | p=0.824 |
|  |  |  |
| **Motor trigeminal nucleus** | **n** | **Total cell bodies per nucleus** |
| Wild type | 9 | 1010.0 ± 38.7 |
| Thy1mγSN | 10 | 668.0 ± 57.9 |
|  |  | p=0.0012 |
|  |  |  |
| **L4/L5 dorsal root ganglion** | **n** | **Total cell bodies per ganglion** |
| Wild type | 32 | 4456.3 ± 150.6 |
| Thy1mγSN | 32 | 4308.2 ± 117.2 |
|  |  | p=0.441 |

**Table A. 1: Motor neurons pools are selectively sensitive to γ-synuclein toxicity whereas sensory neurons are resistant.** For cranial nuclei, left and right nuclei were quantified separately in each animal. For dorsal root ganglia, left and right ganglia for both lumbar 4 and 5 were quantified separately. n=total number of nuclei assessed (mean±SEM, Mann-Whitney U-test).

| **Protein** | **Wild type** | **Thy1mγSN**  **(Mild)** | **Thy1mγSN**  **(Severe)** |
| --- | --- | --- | --- |
| **Neurofilament-H** | 100 ± 11.2 | 79 ± 7.4 | 34 ± 5.4** |
| **Neurofilament-M** | 100 ± 2.5 | 83 ± 11.6 | 45 ± 15.3* |
| **Neurofilament-L** | 100 ± 10.3 | 72 ± 9.6 | 36 ± 6.1** |
| **Peripherin** | 100 ± 23.8 | 87 ± 2.2 | 76 ± 17.6 |
| **Alpha-tubulin** | 100 ± 11.7 | 89 ± 6.2 | 42 ± 10.2* |
| **Actin** | 100 ± 10.4 | 112 ± 9.2 | 105 ± 7.8 |
| **Myelin Basic Protein (21kDa)** | 100 ± 9.5 | 61 ± 10.0* | 49 ± 7.3** |
| **Myelin Basic Protein (18kDa)** | 100 ± 9.1 | 33 ± 1.4* | 18 ± 0.6** |

**Table A. 2: Cytoskeletal components are depleted in the sciatic nerves of the most symptomatic Thy1mγSN mice.** Levels of protein markers in the total lysates of the sciatic nerve of 12-month old mice expressed as % of wild type mean after normalization to the level of GAPDH in the same sample (mean±SEM, * p<0.05, ** p<0.01, Mann-Whitney U-test). Data obtained by densitometry of protein bands on Western blots. Sciatic nerves from 3-6 mice per group we analyzed individually. A representative Western blot is shown in the main Figure 2.

| **Sciatic Nerve** | **n** | **Total**  **A-fibres** | **Healthy**  **A-fibres** | **Damaged**  **A-fibres** |
| --- | --- | --- | --- | --- |
| Wild type | 7 | 269.4 ± 14.3 | 264.4 ± 13.9 | 5.1 ± 1.1 |
| Thy1mγSN (mild) | 3 | 275.7 ± 19.2 | 266.4 ± 20.2 | 9.2 ± 2.5 |
|  |  | p=0.9156 | p=0.9789 | p=0.0493 |
| Thy1mγSN (severe) | 7 | 202.2 ± 10.3 | 162.8 ± 10.5 | 39.4 ± 3.9 |
|  |  | p=0.0005 | p=0.0001 | p<0.0001 |
|  |  |  |  |  |
| **Sciatic Nerve** | **n** | **Total**  **C-fibres** | **Encapsulated**  **C-fibres** | **Atypical**  **C-fibres** |
| Wild type | 3 | 576.0 ± 90.6 | 570.1 ± 91.2 | 5.9 ± 5.9 |
| Thy1mγSN (mild) | 3 | 556.0 ± 138.8 | 553.6 ± 139.2 | 2.5 ± 2.4 |
|  |  | p=0.6458 | p=0.6756 | p=0.5997 |
| Thy1mγSN (severe) | 3 | 529.7 ± 45.8 | 308.0 ± 54.7 | 221.8 ± 36.5 |
|  |  | p=0.8605 | p=0.0318 | p<0.0001 |

**Table A. 3: Nerve fibre counts per 100μm^2^ for sciatic nerve.** n=number of nerves from individual animals assessed. (mean±SEM, Mann-Whitney U-test).

| **Dorsal Nerve Root** | **n** | **Total**  **A-fibres** | **Healthy**  **A-fibres** | **Damaged**  **A-fibres** | **Phagocyte**  **nuclei** |
| --- | --- | --- | --- | --- | --- |
| Wild type | 9 | 262.5 ± 10.0 | 258.8 ± 9.8 | 3.7 ± 0.6 | 0.4 ± 0.2 |
| Thy1mγSN | 10 | 254.6 ± 9.7 | 239.0 ± 9.8 | 15.6 ± 1.1 | 4.6 ± 0.8 |
|  |  | p=0.8493 | p=0.2716 | p<0.0001 | p=0.0002 |
|  |  |  |  |  |  |
| **Ventral Nerve Root** | **n** | **Total**  **A-fibres** | **Healthy**  **A-fibres** | **Damaged**  **A-fibres** | **Phagocyte**  **nuclei** |
| Wild type | 9 | 95.8 ± 3.4 | 94.3 ± 3.3 | 1.5 ± 0.4 | 0.1 ± 0.1 |
| Thy1mγSN | 10 | 78.6 ± 3.9 | 62.5 ± 3.1 | 16.2 ± 1.9 | 8.4 ± 1.2 |
|  |  | p=0.0005 | p<0.0001 | p<0.0001 | p<0.0001 |

**Table A. 4: Nerve fibre counts per 100μm^2^ for dorsal and ventral nerve roots.** n=number of nerves from individual animals assessed. (mean±SEM, Mann-Whitney U-test).

| **Optic Nerve** | **n** | **Total A-fibres** | **Mean fibre area** | **g-ratio** |
| --- | --- | --- | --- | --- |
| Wild type | 3 | 4307.2 ± 138.4 | 1.86 ± 0.1 | 0.51 ± 0.01 |
| Thy1mγSN | 3 | 3975.3 ± 143.5 | 1.90 ± 0.07 | 0.50 ± 0.00 |
|  |  | p=0.0750 | p=0.9398 | p=0.262 |

**Table A. 5: Optic nerve fibres counts per 100μm^2^.** n=number of nerves from individual animals assessed (Mean±SEM, Mann-Whitney U-test).
